# Supplementary material for: Impaired neuronal sodium channels cause intranodal conduction failure and reentrant arrhythmias in human sinoatrial node
Source: Nat Commun. 2020 Jan 24;11:512. doi: 10.1038/s41467-019-14039-8 (PMC6981137; doi:10.1038/s41467-019-14039-8)
Supplement: Supplementary file 1 — Supplementary Information [file 41467_2019_14039_MOESM1_ESM.pdf]

Impaired neuronal sodium channels cause intranodal conduction failure and reentrant arrhythmias in human sinoatrial node

Li et al

SUPPLEMENTAL MATERIALS

Impaired neuronal sodium channels cause intranodal conduction failure and reentrant arrhythmias in human sinoatrial node

Authors: Ning Li[1,2], Anuradha Kalyanasundaram[1,2], Brian J. Hansen[1,2], Esthela J. Artiga[1], Roshan Sharma[3], Suhaib H. Abudulwahed[1], Katelynn M. Helfrich[1,2], Galina Rozenberg[1], Pei-Jung Wu, Stanislav Zakharkin[1], Sandor Gyorke[1,2], Paul ML. Janssen[1,2], Bryan Whitson[2,4], Nahush A Mokadam[2,4], Brandon J. Biesiadecki[1,2], Federica Accornero[1,2], John D. Hummel[2,5], Peter J. Mohler[1,2], Halina Dobrzynski[6,7], Jichao Zhao[3] & Vadim V. Fedorov[1,2]

Affiliations: [1]Department of Physiology & Cell Biology; [2] Bob and Corrine Frick Center for Heart Failure and Arrhythmia, Davis Heart & Lung Research Institute; The Ohio State University Wexner Medical Center, Columbus, OH, USA. [3]Auckland Bioengineering Institute, The University of Auckland, Auckland, New Zealand. [4]Department of Surgery. [5]Department of Internal Medicine, The Ohio State University Wexner Medical Center, Columbus, OH, USA. [6]Division of Cardiovascular Sciences, The University of Manchester, Manchester, UK. [7]Department of Anatomy, Jagiellonian University Medical College, Cracow, Poland.

SUPPLEMENTARY METHODS

Explanted human hearts with intact SAN pacemaker complexes were obtained from The Ohio State University Cardiac Transplant Team and LifeLine of Ohio Organ Procurement Organization in accordance with The Ohio State University Institutional Review Board. Human SAN and atrial tissue was utilized for optical mapping experiments (n=14, Supplementary Table I) or multi-regional molecular study (n=20, Supplementary Table II). Six-digit case numbers are presented in parentheses in the figure and figure legends in which they appear.

Optical Mapping of Coronary-Perfused Human Atrial Preparations

Explanted human hearts were arrested and cooled to 4°C in the operating room following cross-clamping of the aorta. Hearts were stored in cold cardioplegic solution (4°C) during transport, dissection, and cannulation. Human atrial preparations (n=10) were isolated, coronary-perfused, and superfused with 36.5±0.5°C oxygenated Tyrode's solution under constantly maintained pH (7.35±0.05) and pressure (55±5 mmHg)[1, 2]. All preparations excluded regions of poor coronary perfusion/ischemia. After 40-70min of washout and warming to 37°C with oxygenated Tyrode's solution to ensure tissue recovery and stabilization, the preparations were immobilized with 10µM blebbistatin (Abcam) and stained with near-infrared dye di-4-ANBDQBS (10-40µM, University of Connecticut)[1-3]. Isoproterenol bolus (0.2 mL of 1µM, Sigma-Aldrich) was used in some experiments to help recover sinus rhythm during first 10-40 minutes of the experiments when SAN preparations were acclimatized to warm Tyrode solution from the ice-cold cardioplegic solution. During these first minutes of coronary perfusion SAN preparations may have depressed automaticity and adding bolus of isoproterenol quickly restores stable pacemaker and conduction function and facilitated washout from cardioplegic solution from interstitial compartments. Additionally, if several drug protocols were studied, then the first drug studied was washed out from SAN preparations, and isoproterenol 1-10 nM may have been used to recover sinus rhythm to it baseline levels before the effect of Nav blockade was studied (Supplementary Table 6-7).

Imaging was simultaneously conducted with two to four MiCAM Ultima-L CMOS cameras (SciMedia, Ltd., CA USA) with optical field-of-view 3.3x3.3cm^2 (330μm^2 resolution, 100×100 pixels) sampled at 1000 frames/s. The preparations were instrumented with two customized bipolar pacing electrodes placed on the right atrial epicardial surface. Additionally, a far-field pseudo atrial ECG was recorded by two Ag–AgCl plaque electrodes (9mm diameter).

Following motion suppression and dye staining, preparations were equilibrated for 20–30min before imaging. SAN preparations were imaged during sequential perfusion of regular Tyrode's solution (control, n=10), 100nM (n=8), and/or 1-3μM (n=7) tetrodotoxin (TTX, Abcam). Effects of TTX were assessed 15-25 minutes after administration. The time interval between drug doses was 30-40min. After sinus rhythm recordings, all preparations were paced at a cycle length of 500ms to evaluate the direct and indirect sinus node recovery time (SNRT*d*/SNRT*i*)[1-4], and paced incrementally until the functional refractory period was reached[3-6]. Adenosine boluses (1 mL of 10 μM, 30 μM, and 100 μM, Sigma-Aldrich) were injected through coronary perfusion to challenge the robustness of SAN pacemaking and conduction. Pacing and adenosine protocols were repeated for each drug dose.

Electrophysiology Data Analysis

All optical mapping data were analyzed using a custom-made Matlab program as previously described[1-4]. Intramural SAN signals were extracted from background atrial signals as previously described[4]. Analysis of optical action potential morphology and reconstruction of activation patterns allowed for the identification of the leading pacemaker, or area of earliest SAN depolarization, as well as areas of earliest atrial activation, where SAN activation exited the SAN and activated atrial myocardium through SAN conduction pathways (SACPs). SAN conduction time (SACT) was measured during sinus rhythm (SACT*sr*) and the first post-pacing SAN beat (SACT*ppb*). SNRT*i* was calculated as the interval from the last paced atrial beat to the first post-pacing atrial beat, which is the traditional method for SNRT measurement in the clinical setting.

SNRT*d* was calculated as the interval from the last paced atrial beat to the first spontaneous SAN activation measured by optical mapping[7]. Corrected SNRT (cSNRT*i/d*) was calculated by subtracting the preceding sinus cycle length (SCL) from SNRT. SAN and atrial activation patterns were analyzed during control, and TTX perfusion. In hearts with atrial arrest, the SCL, SACT, and SNRT were measured when the spontaneous sinus rhythm recovered. SACT during SAN exit block was measured as the full SAN conduction time as described previously[8].

Histology, PCR, immunoblotting, and immunostaining

To confirm the anatomical location of the SAN and SACPs of optically mapped preparations, the SAN tissue was sectioned for Masson's trichrome staining and immunostaining as described elsewhere[8-10]. Shortly after optical mapping, the SAN pacemaker complex was formalin-fixed, paraffin-embedded, and serial sectioned. Histological sections at 5µm thick were stained with Masson's trichrome (Sigma Aldrich) and sister sections were immuno-labeled with connexin-43 (Cx43, Sigma Aldrich c-6219) and α-actinin (Sigma Aldrich, A-7811). Based on our previous studies on the human SAN[2, 10], we identified the major components of the SAN complex including the head, center, and tail pacemaker compartments, as well as the five main SACPs (lateral superior/middle/inferior, superior vena cava, and septal pathways)[2, 9-11].

To molecularly map the mRNA and protein distribution of neuronal and cardiac Nav isoforms, the human SAN complex and surrounding atrial tissue from 20 unmapped hearts (10 heart failure and 10 non-failing) were embedded in OCT (Fisher) and frozen in liquid nitrogen. Based on anatomic data, frozen SAN tissues (cryoblocks) were cut perpendicularly to the epicardium into head, center, and tail blocks (~4-6 mm thick) [2, 9]. Cryosections were collected from both ends of the cryoblocks at 16µm thickness. Masson's trichrome staining for fibrotic tissue and Cx43/α-actinin double immunolabeling were performed on cryo slides to guide SAN tissue collection from cryoblocks by 16G (1.3mm I.D.) biopsy needles[9] (Supplementary Fig 12). Cx43 negative areas

of α-actinin positive region near SAN artery were identified as pure SAN pacemaker tissue. Total RNA was extracted[12] from the SAN and surrounding atrial tissue separately.

Quantitative polymerase chain reaction (qPCR), and immunofluorescence were performed as previously described[9, 12] to investigate the molecular distribution of Nav isoforms. Quantitative PCR was conducted with QuantStudio 3 (Applied Biosystems), SYBR green (Qiagen), and QuantiTect primer assays (Qiagen; Supplementary Table 8). The ribosomal 18s reference gene was used to normalize the data and comparative threshold cycle (Ct) was used to compare the relative abundance of mRNAs in the samples. Primary antibodies against Nav1.5 (Custom-made in Dr. Mohler's lab)[13], Nav1.6 (Supplementary Table 9)[14], Cx43, and α-actinin were used to quantify corresponding proteins by immunoblotting and immunostaining [2, 9]. Vimentin and tyrosine hydroxylase were used to label fibroblasts and nerve bundles[9, 15] respectively.

Two-dimensional SAN-SACP-RA model

The 2D computer SAN-SACP-RA model used in this study was based on a simplified representation of our histologically reconstructed 3D geometry of the human SAN[10] by considering the relative larger size of atria to the SAN complex and SACPs. In our geometrical model, the SAN has 30 x 15 cells and the SACP has 14 x 3 cells; in contrast, the atrium has 56 x 31 cardiac cells. The SAN and SACP cells were modeled by adapting the Fabbri et al. model[16] to reproduce optical mapping data (Supplementary Table 10). The RA cells were modeled by using the original Courtemanche et al. model[17]. All computer simulations were run parallel on the New Zealand eScience Infrastructure (NeSI) and post-processed using the high-performance computing system at the Auckland Bioengineering Institute, The University of Auckland, New Zealand. Transmembrane voltages and individual ionic currents were exported for detailed analysis using Matlab.

Safety factor was utilized to measure the success of propagation at each cell and is defined as the ratio of the total charge produced to the total charge consumed at that cell. If the ratio is less than 1, inefficient charge is produced for downstream activation and propagation will fail. Safety factor was calculated using the same formula used in the study by Shaw and Rudy[18]:

$$SF = \frac{Q_{out} + Q_c}{Q_{in}}$$

The total charge consumed Q_{in} is computed by integrating over a time interval (**A**) of current entering the cell. Here, we chose the lower and upper bound of this time interval **A** as the instant when the membrane potential derivative reaches 1% of its maximum and the instant when membrane potential is maximal, respectively. The term Q_{out}, refers to the total charge passed to neighboring cells. Q_{out} can be computed similarly by integrating current exiting the cell over the same time interval **A**. The capacitive charge of the membrane Q_c, the energy reserved for the membrane repolarization, is equal to the time integral of the membrane capacitive current.

SUPPLEMENTARY TABLES

Supplementary Table 1. Human heart information for optical mapping experiments

Heart No.	Case No.	Diagnoses	Control SCL(ms)	nNav1.6 Blocker SCL(ms)	TTX 100nM SCL (ms)	TTX1-3μM SCL (ms)
**1**	118258	HTN, LV hypertrophy, smoker, chronic alcohol consumption, drug abuse	536			634
**2**	749693	HTN	545			580
**3**	872295	DM	537		565	1085
**4**	799415	DM, HTN, smoker, chronic alcohol consumption, drug abuse	792		915	
**5**	421856	HF, AF	1080		1089	
**6**	397128	Chronic alcohol consumption, CVA	537		602	
**7**	642519	Asthma, smoker, respiratory arrest	944		1011	1184
**8**	930597	HF, HTN, ICD, AF	1080		1068	2188
**9**	228749	HTN	516		543	649
**10**	567093	Cardiac arrest, DM, drug abuse	700		736	831
**11***	957855	CAD, DM, HTN	855	876		
		Washout	$628^{\#}$	675	732	
**12**	784360	Non cardiac disease, chronic alcohol consumption	623	718	885	
**13***	283273	HTN, chronic alcohol consumption	661	684	764	
**14***	670263	Non cardiac disease	897	896	941	

AF indicates atrial fibrillation; CAD, coronary artery disease; CM, cardiomyopathy; CVA, cardiovascular attack; DM, diabetes mellitus; HF, heart failure; HTN, hypertension; ICD, implantable cardioverter-defibrillator; SCL, sinus cycle length; TTX, tetrodotoxin. [#]Heart 11 was tested with and without isoproterenol 1 nM. * indicate hearts used for immunostaining and mRNA analysis after mapping.

Supplementary Table 2. Human heart information for molecular mapping experiments

Heart No.	Case No.	Diagnosis	Chronic alcohol consumption	Smoking	Drug abuse
15	632941	No cardiac disease	Yes	Yes	No
16	785258	No cardiac disease	Yes	Yes	No
17	694855	No cardiac disease	Yes	Yes	Yes
18	712301	HTN, hyperlipidemia	Yes	No	No
19	294050*	Cardiac arrest	Yes	Yes	Yes
20	435578*	No cardiac disease	No	No	Yes
21	754477*#	Acute cardiac damage due to car accident	No	No	No
22	958987#	Cardiac arrest, Enlarged heart	No	Yes	No
23	900500	COPD	No	No	No
24	652357#	No cardiac disease	No	No	No
25	369452	Non-Ischemic HF, CAD, HTN, ICD	Yes	Yes	No
26	645444	Ischemic HF, CAD, HTN, DM, ICD	No	No	No
27	774694	Ischemic HF, MI, HTN	No	No	No
28	674541	HF, HTN, DM, CAD, MI, ICD	No	Yes	No
29	994744*#	Non-Ischemic HF, HTN, AF, ICD, LVAD	No	No	No
30	335581	Non-Ischemic HF, ICD	No	Yes	No
31	378549*	HF, ICD, AF, HTN, HF, MI,	Yes	Yes	No
32	897154*#	HF, ICD, LVAD, HTN, DM	No	Yes	No
33	437819*#	HF, HTN, ICD, LVAD	No	Yes	No
34	682814*	HF, AF, ICD, LVAD	No	Yes	No

All hearts were used for mRNA analysis, * indicate hearts used for immunostaining, and # indicates hearts used for western blotting. AF indicates atrial fibrillation; CAD, coronary disease; COPD, chronic obstructive pulmonary disease; CVA, cardiovascular attack; DM, diabetes mellitus; HF, heart failure; HTN, hypertension; ICD, implantable cardioverter-defibrillator; LAVD, left ventricular assist device; MI, myocardial infarction.

Supplementary Table 3. Relative mRNA abundance in failing and non-failing human SAN and RA

mRNA	SAN (NF) n=10	SAN (HF) n=10	RA (NF) n=10	RA(HF) n=10	Non-chronic alcohol consumers			
					SAN (NF) n=5	SAN (HF) n=8	RA (NF) n=5	RA (HF) n=8
SCN1A	31.9±25.6	44.1±45.2	8.0±8.5	21.6±26.9	25.4±31.4	36.7±44.0	9.45±6.3	24.9±29.2
SCN2A	0.8±0.8	0.8±0.9	0.4±0.2	0.2±0.1*	1.34±0.9	0.87±0.9	0.55±0.2	0.21±0.1*
SCN3A	8.0±4.1	9.7±7.8	8.7±4.9	4.7±4.7*	9.5±4.1	9.1±8.1	9.9±5.6	5.2±5.1*
SCN4A	0.4±0.4	0.3±0.4	1.2±1.1	0.4±0.3*	0.20±0.07	0.33±0.4	1.14±0.7	0.38±0.3*
SCN5A	125±139	85±57	379±183	270±79	70.2±55.1	80.6±54.7	390±174	254±55
SCN8A	1.2±0.8	1.0±0.5	0.5±0.4	0.5±0.2	1.73±0.6	1.03±0.4*	0.64±0.5	0.47±0.2*
SCN9A	8.5±2.8	8.2±7.8	3.2±1.6	1.9±1.4*	9.33±2.6	8.4±8.5	3.7±2.1	1.88±1.5
SCN11A	0.1±0.1	0.2±0.1	0.1±0.1	0.2±0.1	0.11±0.06	0.19±0.1	0.15±0.1	0.18±0.1
SCN1B	37±25	42±43	362±253	177±95	26.1±12.4	28.4±21.1	273.6±151	161±66.4
SCN2B	4.8±2	6.4±3.5	18.2±7.8	11.8±4.0*	3.6±1.5	5.4±3.0	18.7±8.5	12.0±4.0*
SCN3B	1.7±1.2	2.3±2.9	2.4±1.6	1.7±0.9	2.1±1.2	2.2±3.1	3.3±1.6	1.7±1.0*
SCN4B	2.7±1.3	3.6±2.0	8.7±4.9	4.6±3.1*	2.1±0.8	3.0±1.8	7.9±4.1	4.1±2.8*
GJA1	98.5±71.2	112.3±19.9	618.5±384.8	307.3±111.1	71.7±34.7	111.7±56.5	821.6±419.4	328.0±113.8*
GJA5	53±42.4	67.1±55.5	155.7±127.3	180.4±145.1	42.6±19.5	65.8±55.4	196.4±172.5	198.7±158.5
HCN1	100.1±79.1	41.4±33.4	46.3±33.1	38.8±20.4	88.1±64.6	33.4±17.4	49.4±37.7	43.5±20.7
HCN4	159.3±82.0	110.5±69.2	108.8±33.1	145.5±175.1	165.4±90.5	98.0±65.7	105.2±67.3	79.1±33.3
CACNA1C	104.2±49.9	94.8±37.8	117.1±81.6	153.5±147.2	106.2±65.8	84.3±33.0	111.5±58.7	164.9±162.6
CACNA1D	19.3±14.0	13.7±13.0	5.6±4.4	5.1±4.5	18.0±14.2	10.7±8.0	6.2±3.3	4.9±4.9
CACNA1G	13.5±7.4	9.7±6.2	11.9±13.2	19.8±18.8	12.4±7.1	7.4±3.6	9.8±8.1	18.5±18.6

Data were calculated by $2^{(-\Delta CT)}$ x10^6 and presented as mean ± standard deviation. **P*-value < 0.05 between heart failure (HF) vs. non-failing (NF) within the same tissue type. Statistical analysis was done using mixed models in package lme4 with patient, heart weight, and indicator

variables for alcohol, smoking, drug abuse, and HF. Patient was considered a random effect, others as fixed effects. RA indicates right atrium; SAN, sinoatrial node. Source data are provided as a Source Data file.

Supplementary Table 4. P-values for the association of disease with Nav mRNA in human SAN and RA

	Heart weight	HF n=10	HTN n=9	AF n=5
*SCN1A*_RA	**0.010**	0.840	0.724	0.767
*SCN1A*_SAN	0.243	0.932	0.887	0.428
*SCN2A*_RA	0.512	**0.020**	0.068	0.614
*SCN2A*_SAN	0.250	0.423	0.912	0.946
*SCN3A*_RA	0.829	**0.021**	0.913	0.663
*SCN3A*_SAN	0.187	0.899	0.547	0.553
*SCN4A*_RA	0.414	**0.032**	0.477	0.569
*SCN4A*_SAN	0.268	0.410	0.377	0.135
*SCN5A*_RA	0.217	0.256	0.824	0.423
*SCN5A*_SAN	0.765	0.238	0.245	0.674
*SCN8A*_RA	0.080	0.147	0.879	0.702
*SCN8A*_SAN	0.541	0.299	0.925	0.144
*SCN9A*_RA	0.680	**0.041**	0.976	0.205
*SCN9A*_SAN	0.161	0.308	0.570	0.913
*SCN11A*_RA	**0.002**	0.445	0.142	0.065
*SCN11A*_SAN	**0.015**	0.870	0.375	0.789
*SCN1B*_RA	0.272	0.094	0.979	0.533
*SCN1B*_SAN	0.506	0.944	0.185	0.359
*SCN2B*_RA	0.656	**0.018**	0.482	0.596
*SCN2B*_SAN	0.206	0.549	0.337	0.560
*SCN3B*_RA	0.814	0.200	0.615	0.780
*SCN3B*_SAN	**0.027**	0.517	0.483	0.563
*SCN4B*_RA	0.492	**0.040**	0.582	0.521
*SCN4B*_SAN	0.113	0.468	0.146	0.561

Statistical analysis was done using mixed models in package lme4 with patient, heart weight, and indicator variables for heart failure (HF), atrial fibrillation (AF) or hypertension (HTN). Patient was considered a random effect, others as fixed effects. RA, right atrium; SAN, sino-atrial node.

Supplementary Table 5. P-values for the association of risk factors with Nav mRNA in non-failing human SAN and RA

Nav mRNA	Alcohol n=5	Smoking n=5	Drug abuse n=3
*SCN1A*_RA	0.561	0.910	0.236
*SCN1A*_SAN	0.499	0.119	0.893
*SCN2A*_RA	0.068	0.942	0.072
*SCN2A*_SAN	**0.026**	0.548	0.375
*SCN3A*_RA	0.093	0.583	0.672
*SCN3A*_SAN	0.204	0.812	0.651
*SCN4A*_RA	0.965	0.367	0.376
*SCN4A*_SAN	0.313	0.909	0.089
*SCN5A*_RA	0.887	0.892	0.454
*SCN5A*_SAN	0.313	0.862	0.379
*SCN8A*_RA	**0.000**	0.877	0.350
*SCN8A*_SAN	**0.000**	0.400	0.404
*SCN9A*_RA	0.369	0.923	0.327
*SCN9A*_SAN	0.112	0.265	0.896
*SCN11A*_RA	0.219	0.252	0.600
*SCN11A*_SAN	0.983	0.092	0.575
*SCN1B*_RA	0.285	0.128	0.738
*SCN1B*_SAN	0.276	0.806	0.507
*SCN2B*_RA	0.691	0.466	0.516
*SCN2B*_SAN	**0.019**	0.931	0.485
*SCN3B*_RA	**0.033**	0.648	0.190
*SCN3B*_SAN	**0.047**	0.886	0.942
*SCN4B*_RA	0.711	**0.041**	0.674
*SCN4B*_SAN	0.145	**0.026**	0.459

Statistical analysis was done using mixed models in package lme4 with patient, heart weight, and indicator variables for alcohol, smoking, or drug abuse. Patient was considered a random effect, others as fixed effects. RA, right atrium; SAN, sinoatrial node.

Supplementary Table 6. Effects of neuronal Nav blockade on human SAN and atrial conduction with and without isoproterenol

	**100nM TTX without Iso**			**100nM TTX with Iso**			
	Percent of Control	SD	n	Percent of Control	SD	n	P value
SCL	105%	6%	6	116%	14%	6	0.1213
SACT*sr*	288%	247%	4	174%	42%	2	0.4274
2Hz SACT*ppb*	310%	97%	3	329%	213%	3	0.8991
2Hz cSNRT*i*	233%	215%	5	130%	199%	6	0.251
2Hz cSNRT*d*	90%	36%	3	183%	279%	3	0.625

Abbreviations: CV, conduction velocity; cSNRT*d/i*, corrected direct/indirect sinus node recovery time; Iso, isoproterenol 1-10nM; RA, right atrial; SACT*sr/ppb*, sinoatrial conduction time at sinus rhythm/ post pacing beat; SAN, sinoatrial node; SCL, sinus cycle length. Percent data are reported as mean and standard deviation (SD). Normality assumption was verified using Shapiro-Wilk test. Parametric data were analyzed with two-sided t-test. Non-parametric data were analyzed with two-sided Wilcoxon test.

Supplementary Table 7. Effects of neuronal and cardiac Nav blockade on human SAN and atrial conduction with and without isoproterenol

	1-3µM TTX without Iso			1-3µM TTX plus Iso			
	Percent of Control	SD	n	Percent of Control	SD	n	P value
SCL	149%	47%	3	138%	43%	4	0.7698
SACTsr	515%	468%	4	533%	382%	3	0.6507
2Hz SACTppb	434%	370	2	390%	280%	3	0.9014
2Hz cSNRTi	2197%	N/A	1	139%	118%	3	N/A
2Hz cSNRTd	78%	127%	2	86%	128%	2	0.3298
SAN CV	48%	34%	3	60%	44%	2	0.7852

Abbreviations: CV, conduction velocity; cSNRT*d/i*, corrected direct/indirect sinus node recovery time; Iso, isoproterenol 1-10nM; RA, right atrial; SACT*sr/ppb*, sinoatrial conduction time at sinus rhythm/ post pacing beat; SAN, sinoatrial node; SCL, sinus cycle length. Percent data are reported as mean and standard deviation (SD). Normality assumption was verified using Shapiro-Wilk test. Parametric data were analyzed with two-sided t-test. Non-parametric data were analyzed with two-sided Wilcoxon test.

Supplementary Table 8. Qiagen QuantiTect primer assays used for qPCR

	Target mRNA	Qiagen QuantiTect primer assay number
Housekeeper:	18s	QT00199367
Voltage-gated Na+ channels:	*SCN1A* (Nav1.1)	QT01008007
	SCN2A (Nav1.2)	QT00070707
	SCN3A (Nav1.3)	QT00064981
	SCN4A (Nav1.4)	QT00009765
	SCN5A (Nav1.5)	QT00091812
	SCN8A (Nav1.6)	QT00020923
	SCN9A (Nav1.7)	QT00001505
	SCN10A (Nav1.8)	QT01008028
	SCN11A (Nav1.9)	QT01016344
	SCN1B (Navβ1)	QT00066080
	SCN2B (Navβ2)	QT00022435
	SCN3B (Navβ3)	QT00002184
	SCN4B (Navβ4)	QT00011802
Voltage-gated Ca^{2+} Channels:	*CACNA1C* (Cav1.2)	QT00053480
	CACNA1D (Cav1.3)	QT00076657
	CACNA1G (Cav3.1)	QT00043043
	CACNA1G (Cav3.2)	QT00075159
	CACNA1I (Cav3.3)	QT00021126
	HCN1	QT00048020
	HCN4	QT00038108
	*GJA5 (*Cx40)	QT00222768
	GJA1 (Cx43)	QT00012684
	ADORA1(A1R)	QT01531635

Supplementary Table 9. Antibodies used for immunoblotting and immunostaining

Antibody name	Lot Number	Catalogue Number	Dilution for immunoblotting	Dilution for immunostaining
Anti-Nav1.5	--------	Custom made	1/500	1/100
Anti-Nav1.6	ASC009AG1340	Alomone ASC-009	--------	1/100
Anti-Connexin43	028M4823V	Sigma C6219	1/4000	1/400
Anti-Connexin43	3018901	MAB3067	--------	1/400
Anti-GAPDH	123M4761V	Sigma G8795	1/10000	--------
Anti-α-actinin	127M4807V	Sigma A7811	1/8000	1/200
Anti-Vimentin	013M4799	Sigma V-6389	--------	1/400
Anti-Tyrosine Hydroxylase	2328305	Millipore AB152	--------	1/200
Goat Anti-Rabbit IgG, Alexa Fluor 568	1832035	Invitrogen A11036	--------	1/400
Goat Anti-Mouse IgG, Alexa Fluor 488	948490	Invitrogen A11001	--------	1/400
Cy3,Goat Anti-Rabbit IgG	110862	Jackson ImmunoRsearch 111-165-144	1/2000	--------
Cy5,Goat Anti-Mouse IgG	123312	Jackson ImmunoRsearch 115-175-146	1/2000	--------

Supplementary Table 10. Parameters of the human SAN and SACP cell models relative to the Fabbri et al. model

Current	SAN cells	SACP cells
I_{Na}	1.0	5.0
I_f	1.0	0.5
I_{CaL}	0.7	1.0
I_{K1}*	0	0.15

These parameters are relative unit free parameters to those used in the original Fabbri et al publication[16]. *The formulation of I_{K1} was taken from the original Courtemanche et al[17] model and added to the Fabbri et al. model. SAN indicates sino-atrial node; SACP, sinoatrial conduction pathway.

SUPPLEMENTARY FIGURES

Supplementary Figure 1. Heart Specific Response to Nav blockade.

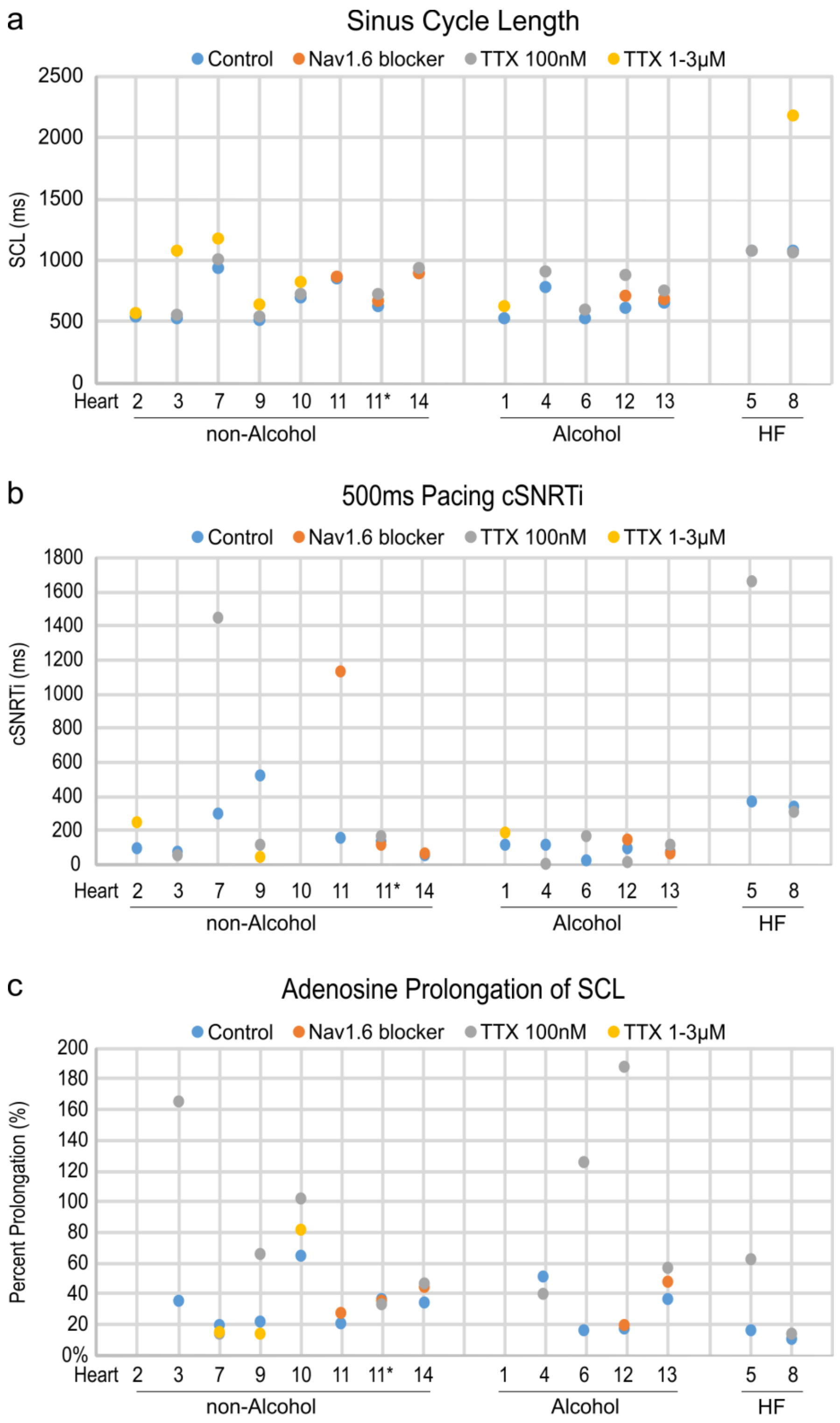

a Sinus cycle length (SCL) values for each condition tested in each heart. **b** Corrected indirect sinoatrial node recovery time (cSNRTi) values for each condition tested in each heart. **c** Percent of SCL prolongation caused by adenosine bolus for each condition tested in each heart. Heart numbers correlate to Supplementary Table 1. Alcohol indicates chronic alcohol consumption; non-Alcohol, no chronic alcohol consumption; HF, heart failure. *Heart 11 shows data without (left) and with (right) isoproterenol 1nM.

Supplementary Figure 2. Poincare plot of heart rate variability

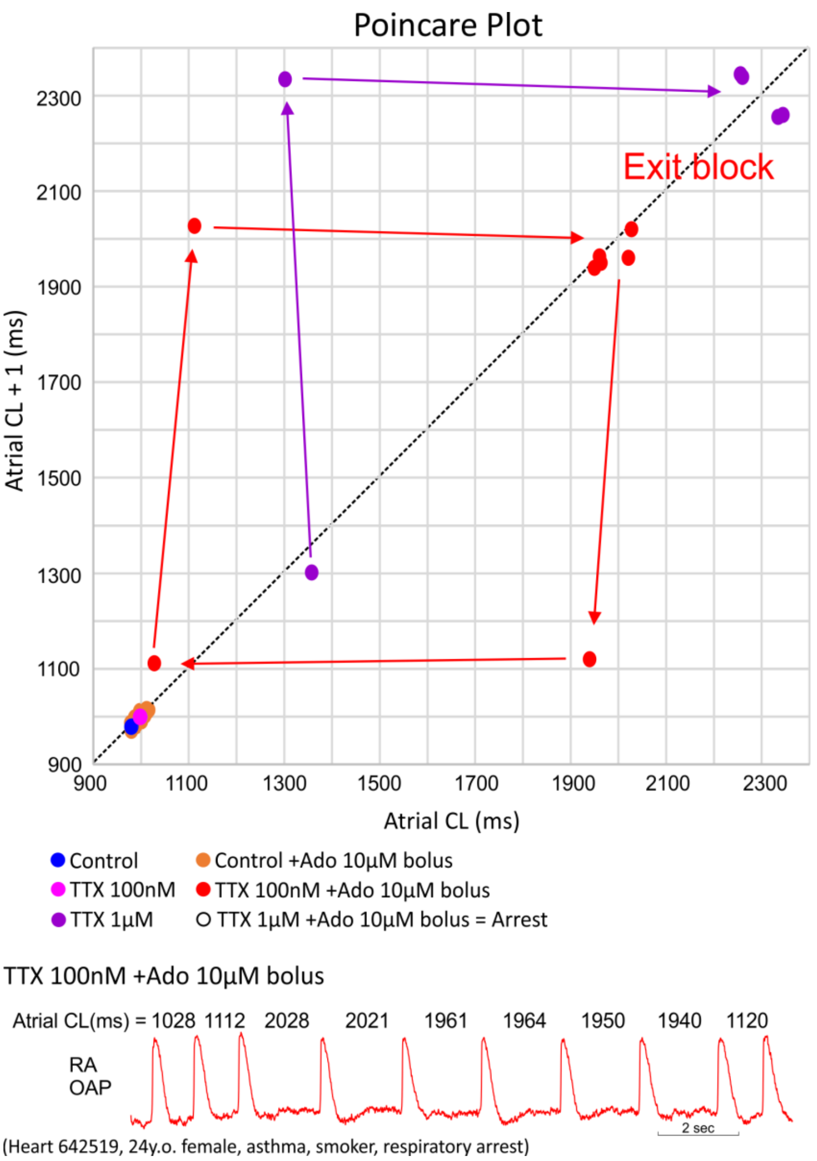

Top, Poincare plot of heart 642519 showing consecutive atrial cycle lengths (CL) plotted against the proceeding cycle length for control, 100nM tetrodotoxin (TTX), 1µM TTX, and adenosine (Ado) bolus during each condition. Ado bolus during 1µM TTX caused complete atrial arrest and thus is not plotted. Line of identity indicated by black dotted line. Bottom, right atrial optical action potential (RA OAP) showing measurements of atrial CL during exit block.

Supplementary Figure 3. Time dependent effect of Sinus rhythm during baseline, nNav1.6 blockade and TTX 100nM in human heart 670263

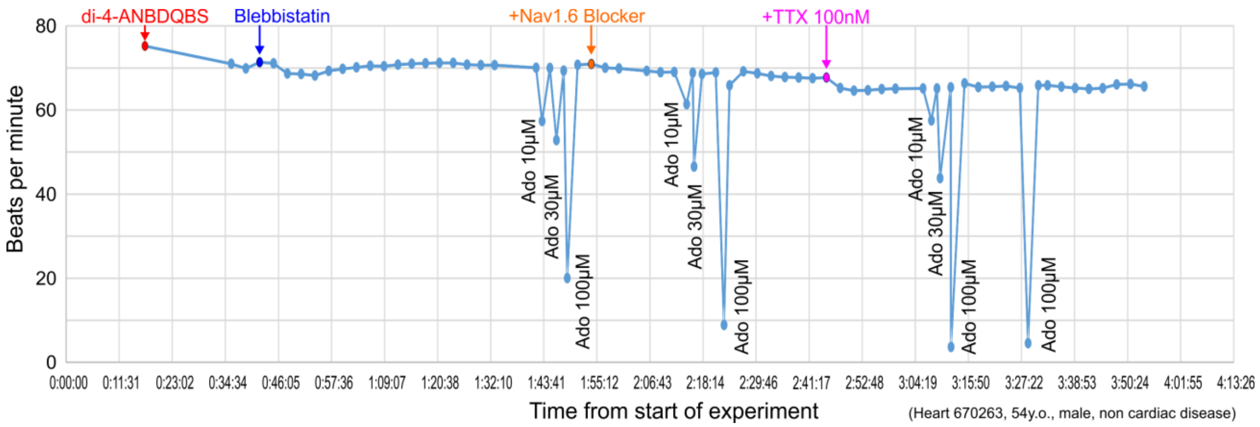

Graph showing beats per minute (BPM) vs time in minutes throughout the 4 hour human coronary-perfused SAN experiment, where both selective nNav1.6 blocker (30 nM) and subsequent tetrodotoxin (TTX) 100nM were added to perfusate. Sharp downward deflections in heart rate represent transient effect of adenosine (Ado) boluses, 10, 30, and 100 µM, added sequentially.

Supplementary Figure 4. nNav1.6 blockade increases SAN conduction impairment caused by adenosine in human heart 957855

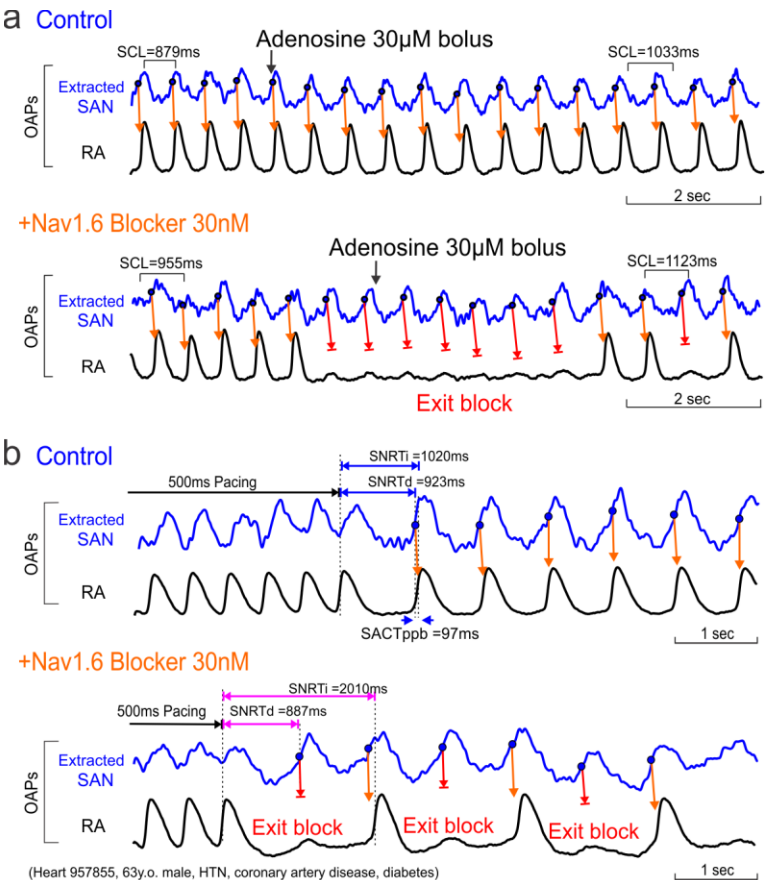

a Optical action potentials (OAPs) showing increase of sinus cycle length (SCL) and sinoatrial node (SAN) exit block due to SAN hyperpolarization with 30µM adenosine bolus at control conditions and under selective neuronal Nav1.6 (nNav1.6) blocker 30nM 4,9-Anhydrotetrodotoxin. **b** OAPs showing reduced SAN recovery from overdrive pacing at control conditions and under selective nNav1.6 blocker. Abbreviations as in Supplementary Fig.1; OAPs, optical action potentials; RA- right atria; SACT*ppb*, sinoatrial conduction time for first post-pacing beat; SNRT*d*, direct sinoatrial node recovery time.

Supplementary Figure 5. Effects of Nav blockade on human atrial conduction

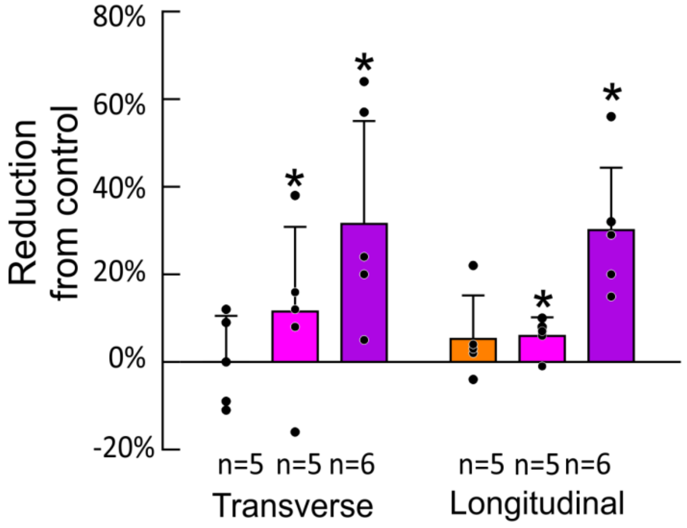

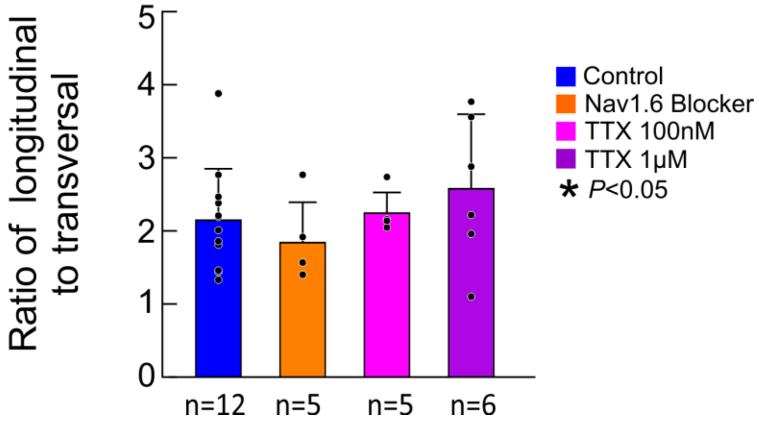

Graphs showing the summary data of tetrodotoxin (TTX) effects on atrial conduction velocity at 500ms pacing. **P*<0.05 compared to baseline. Data are presented as mean ± standard deviation. Statistical analysis was done using *t*-test. Source data are provided as a Source Data file.

Supplementary Figure 6. Antibody specificity

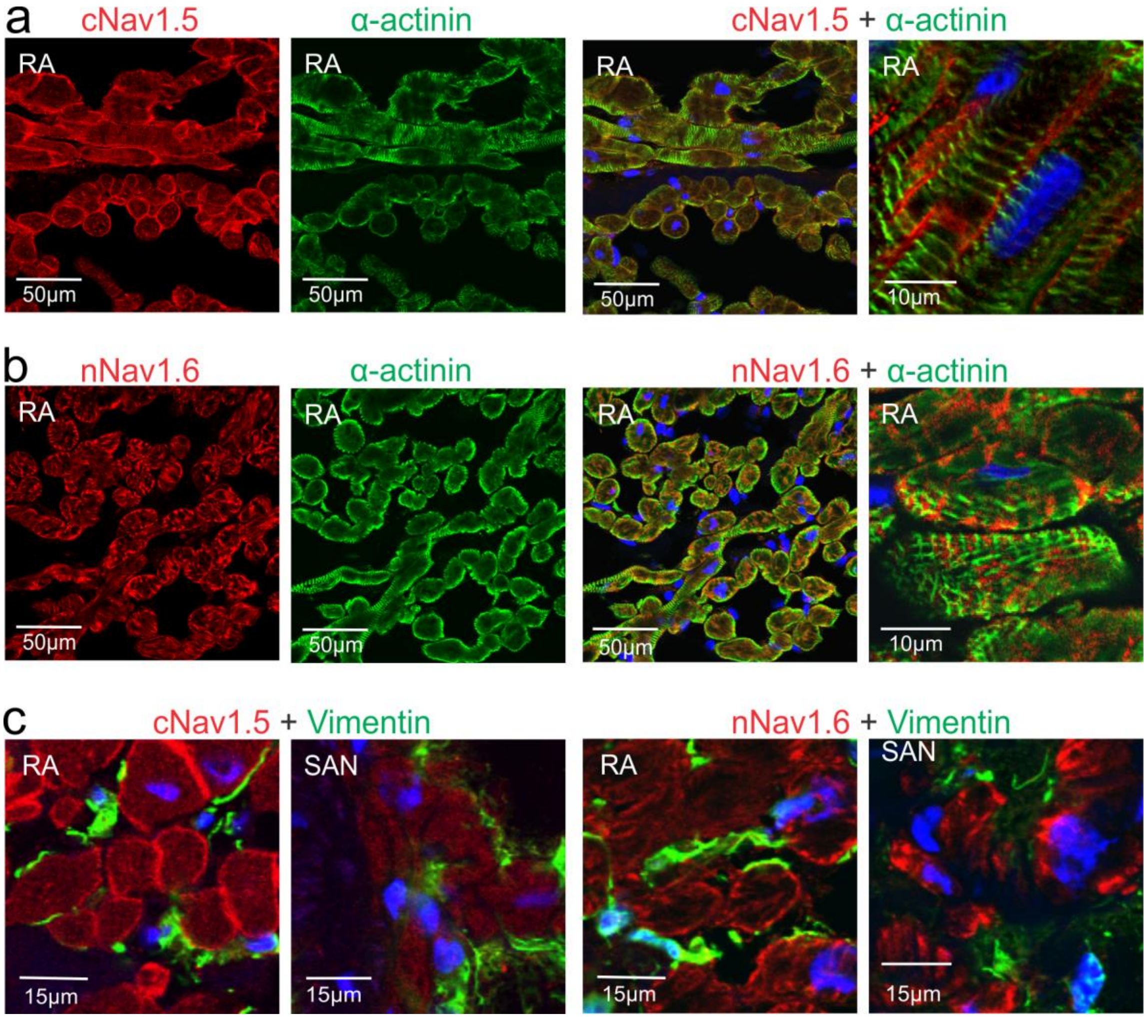

a cNav1.5 (red) and α-actinin (green; staining cardiomyocytes) dual staining; **b** nNav1.6 (red) and α-actinin (green) dual staining confirmed the cardiomyocyte-specific localization of cNav1.5 and nNav 1.6. **c** cNav1.5 (red, left panels) and nNav1.6 (red, right panels) and vimentin (green; staining fibroblasts) dual staining confirmed that these channels are not localized in non-myocytes, including fibroblasts.

Supplementary Figure 7. Quantification of nNav1.6 and cNav1.5 protein expression in 3 functionally mapped human hearts

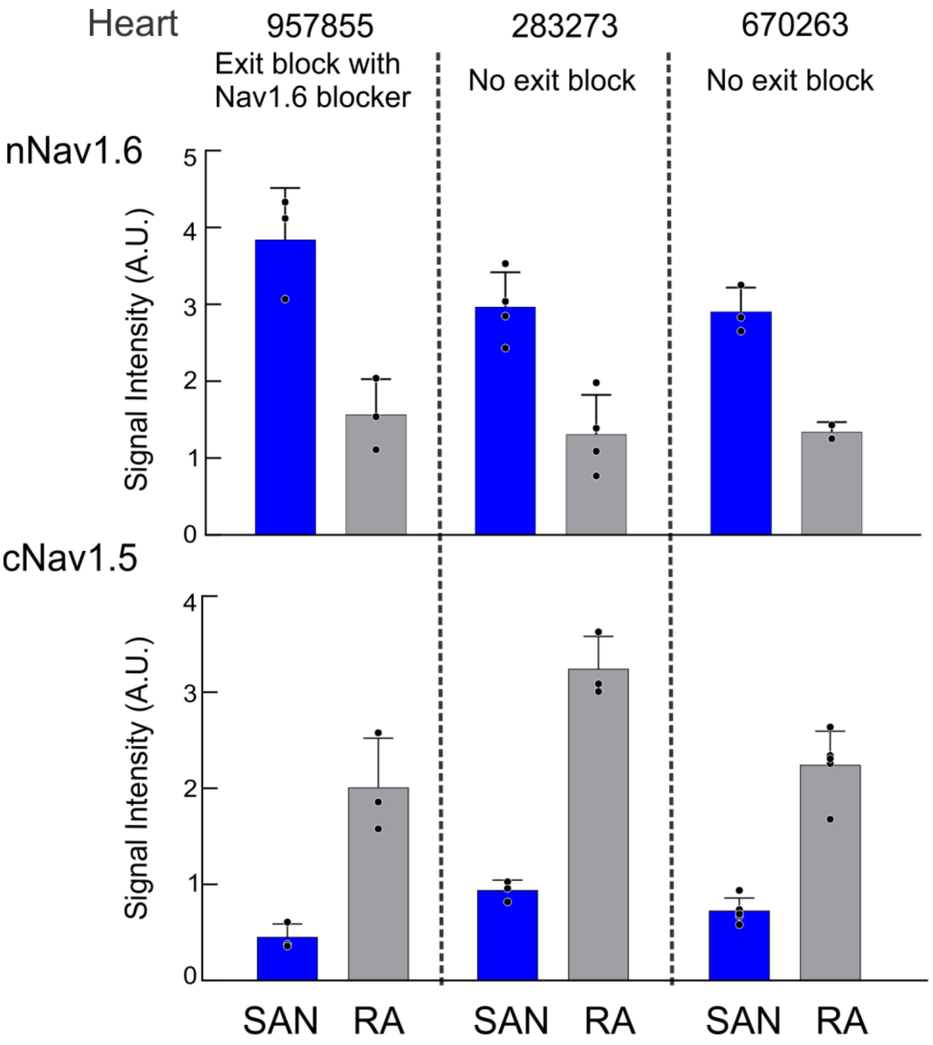

Quantification of nNav1.6 (Top) and cNav1.5 (Bottom) protein expression by immunostaining in 3 functionally mapped hearts (hearts 957855, 283273, and 670263). Signal intensities were averaged from confocal microscopic images (collected at 60X magnification) of immunostained frozen cardiac sections taken after optical mapping. Data presented as average ± standard deviation. Source data are provided as a Source Data file.

Supplementary Figure 8. Nav mRNA levels are correlated with heart weight and significantly altered in smokers

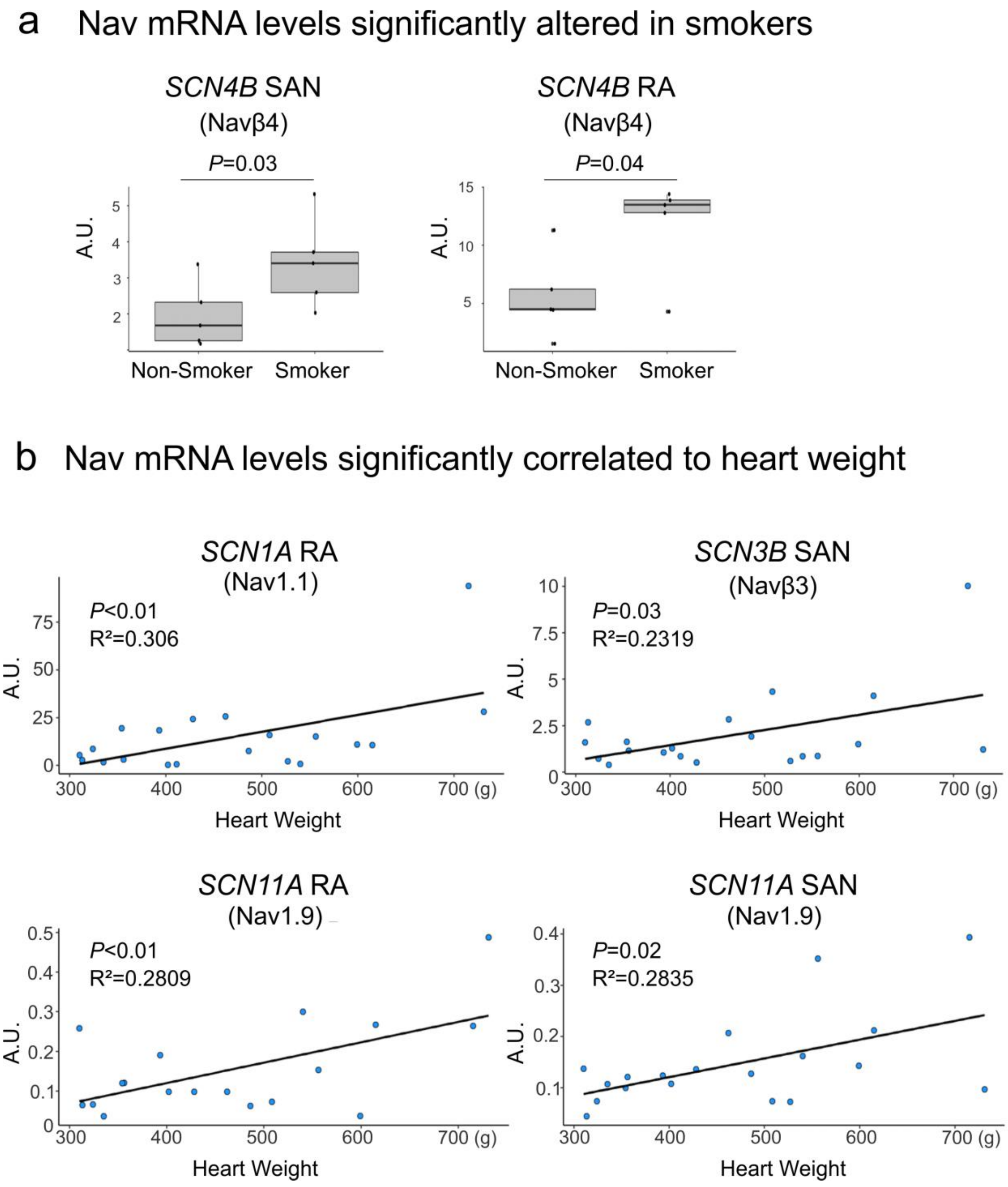

a Nav mRNA levels are significantly altered in smokers. Data distributions are presented as Box plots with dots as individual observations. The horizontal lines inside boxes represent medians. N=5 in each group. **b** Nav mRNA levels correlated to heart weight, n=20. Statistical analysis was done using mixed models in package lme4 with patient, heart weight, and history of smoking. Patient was considered a random effect, others as fixed effects. Source data are provided as a Source Data file.

Supplementary Figure 9. Simulated I_{Na} across the SACP

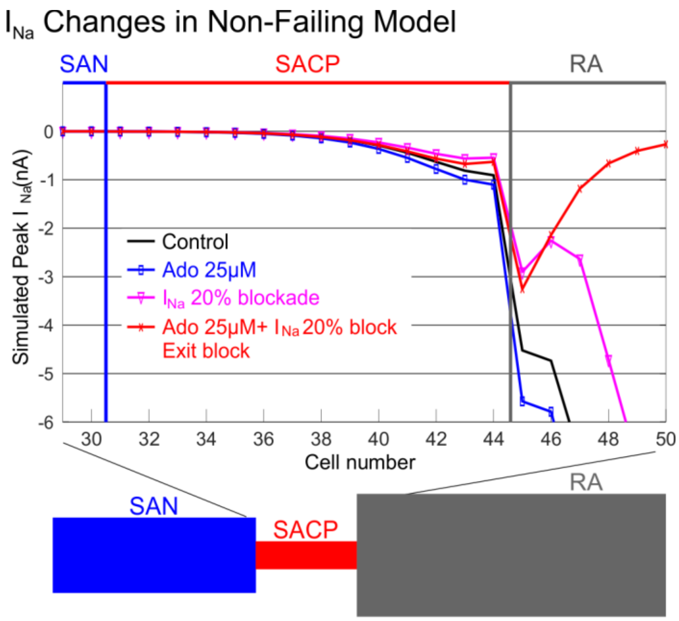

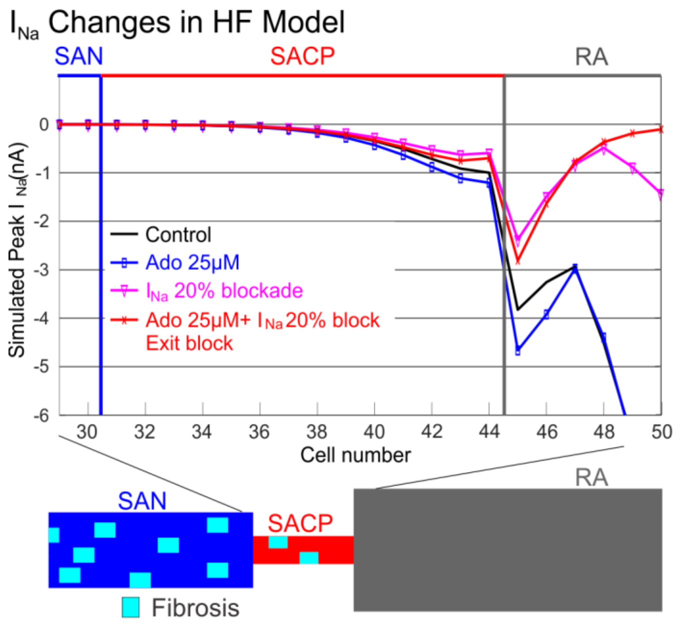

Plot of simulated peak sodium current (I_{Na}) in each cell across the sinoatrial conduction pathway (SACP) in 2D human control (top) and heart failure (HF, bottom) SAN-SACP-RA models; a schematic is shown below. Ado, adenosine; RA, right atrium; SAN, sinoatrial node.

Supplementary Figure 10. Nav blockade in SAN is more detrimental to SAN function than Nav blockade in RA in computer simulations

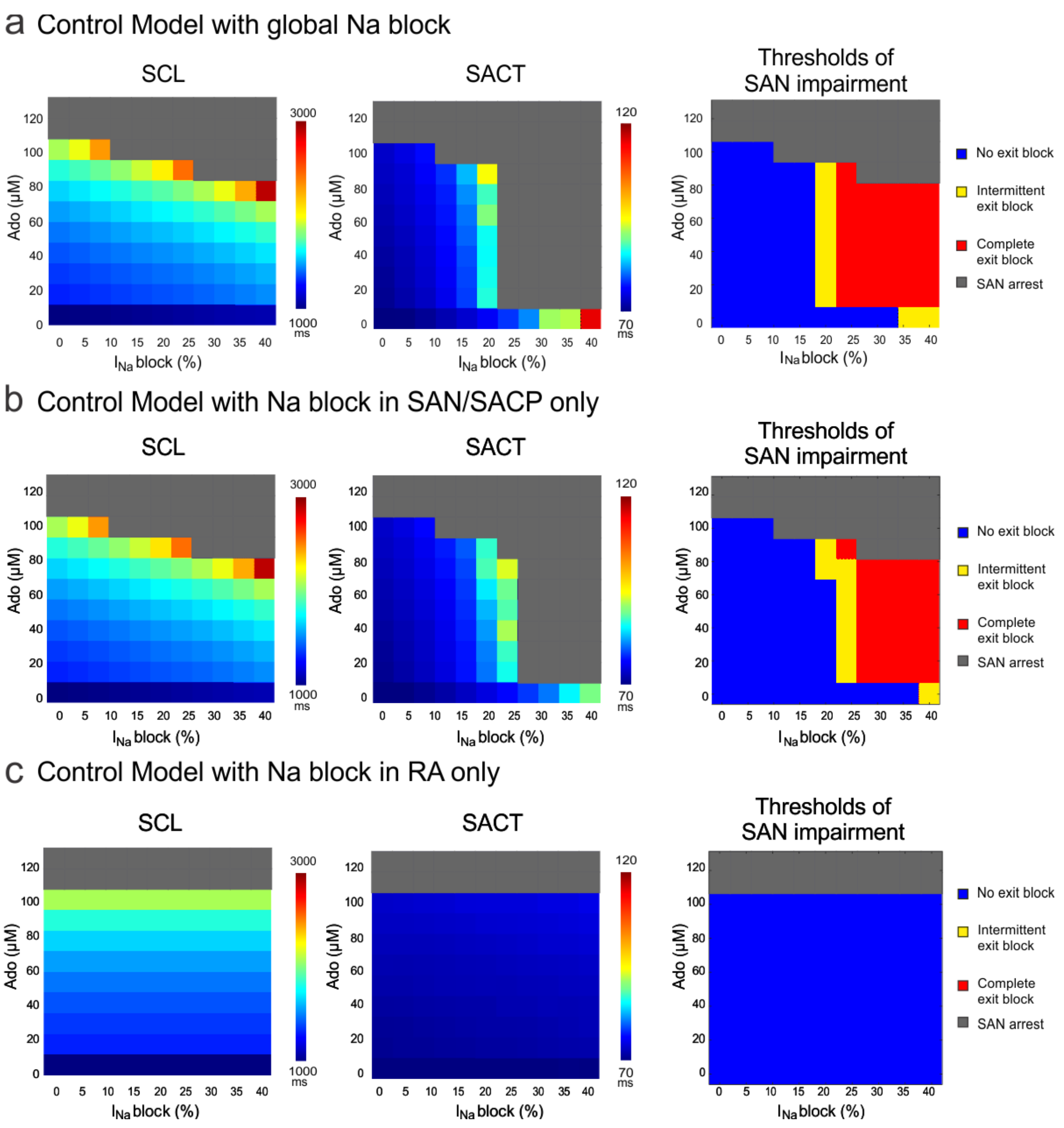

Computer simulation results displaying combinations of adenosine (Ado) dose and the percentage of sodium current (I_{Na}) blockade in terms of sinus cycle length (SCL), sinoatrial conduction time (SACT), and threshold of sinoatrial node (SAN) automaticity and conduction

impairment in control model with Nav blockade simulated globally (**a**), in the SAN/sinoatrial conduction pathway (SACP) only (**b**), and in the right atrium (RA) only (**c**).

Supplementary Figure 11. Atrial pacing suppresses SAN function in computer simulations

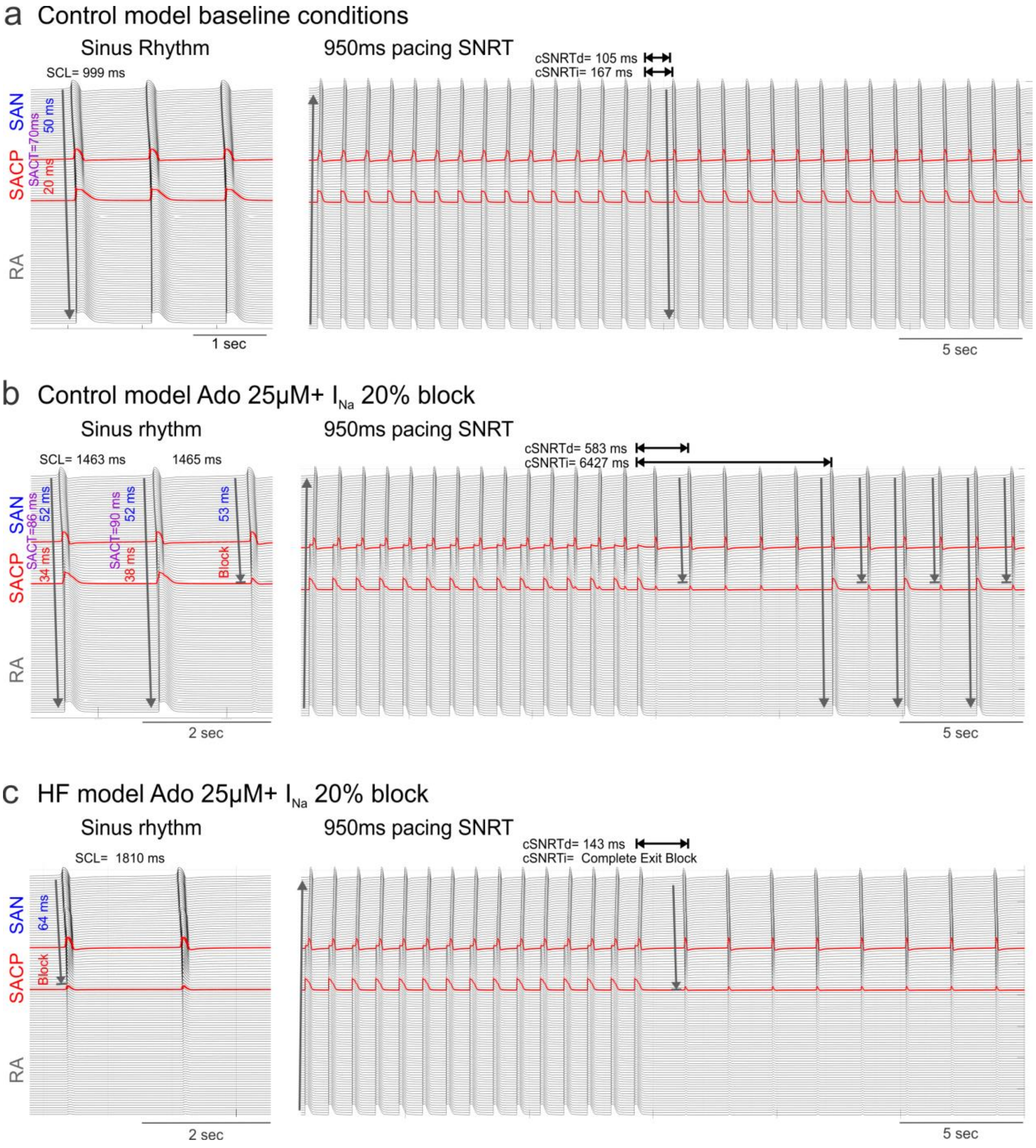

All plots show propagation of APs along the middle axis of the 2D sinoatrial node (SAN)-right atrium (RA) computer model displayed from SAN (top) to RA (bottom). Left plot shows sinus rhythm (SR) while right plot shows sinoatrial node recovery time (SNRT) taken by pacing the atria

at 950ms then stopping pacing midway through the simulation to observe recovery of SAN rhythm. **a** SR and SNRT for control model at baseline. **b** SR and SNRT for control model under 25µM adenosine (Ado) and 20% I_{Na} blockade. **c** SR and SNRT for heart failure (HF) model under 25µM Ado and 20% I_{Na} blockade. Blue and red numbers indicate the conduction time from SAN leading pacemaker through SAN and SACP, respectively. Abbreviations as in Supplementary Fig. 4.

Supplementary Figure 12. Molecular mapping of the human SAN and atria

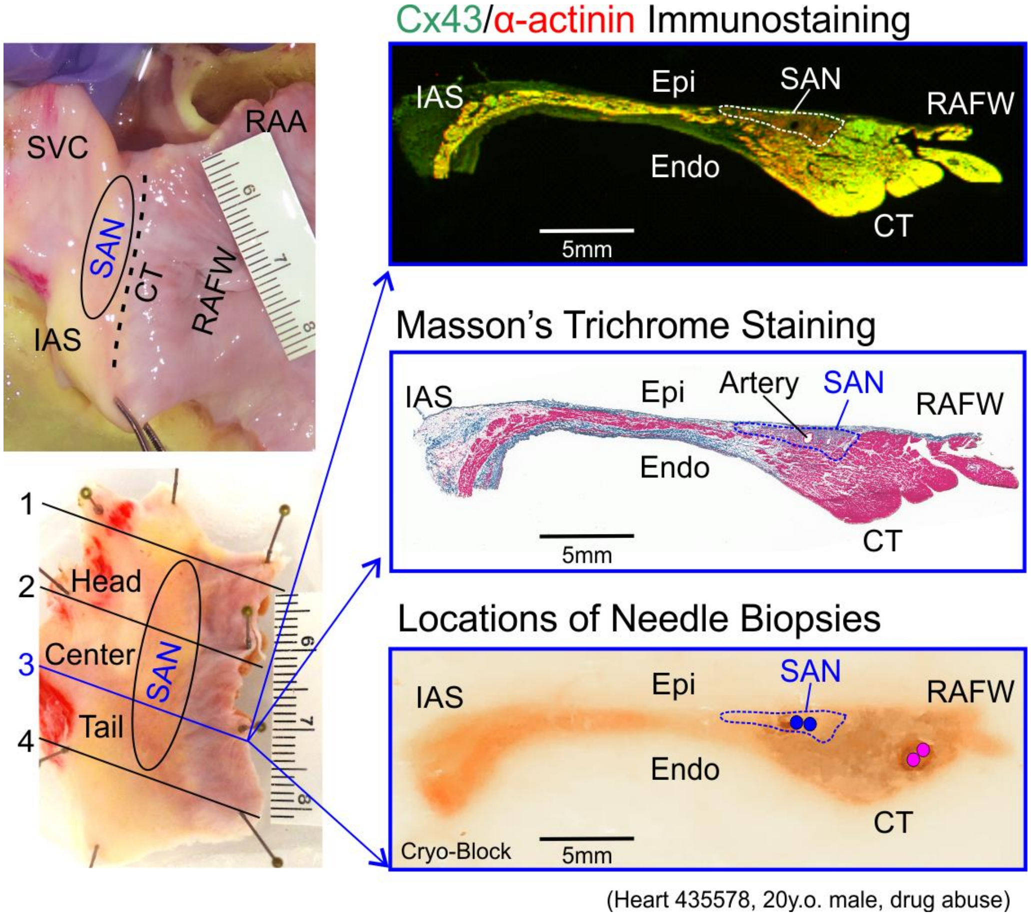

Top left, photograph of human right atrium. Bottom left, human SAN pacemaker-conduction complex pinned to silicon and marked into thirds to dissect the SAN head, center, and tail compartments. Right, Immunostaining (top) and Masson's trichrome histology (middle) sections were used to demarcate SAN borders and guide biopsy extraction (dots) of pure SAN (blue) and atrial (pink) tissue from cryo-blocks (bottom). CT, crista terminalis; Cx43, connexin 43; Endo/Epi, endocardium/epicardium; IAS, interatrial septum; RAA, right atrial appendage; RAFW, right atrial free wall; SAN, sinoatrial node; SVC, superior vena cava.

SUPPLEMENTARY REFERENCE

1. Fedorov,V.V. *et al.* Optical mapping of the isolated coronary-perfused human sinus node. *J Am. Coll. Cardiol.* 56, 1386-1394 (2010).
2. Li,N. *et al.* Redundant and diverse intranodal pacemakers and conduction pathways protect the human sinoatrial node from failure. *Sci. Transl. Med.* 9, (2017).
3. Li,N. *et al.* Adenosine-Induced Atrial Fibrillation: Localized Reentrant Drivers in Lateral Right Atria due to Heterogeneous Expression of Adenosine A1 Receptors and GIRK4 Subunits in the Human Heart. *Circulation* 134, 486-498 (2016).
4. Lou,Q. *et al.* Tachy-brady arrhythmias: The critical role of adenosine-induced sino-atrial conduction block in post-tachycardia pauses. *Heart Rhythm* 10, 110-118 (2013).
5. Fedorov,V.V. *et al.* Effects of KATP channel openers diazoxide and pinacidil in coronary-perfused atria and ventricles from failing and non-failing human hearts. *J. Mol. Cell Cardiol.* 51, 215-225 (2011).
6. Hansen,B.J. *et al.* Atrial fibrillation driven by micro-anatomic intramural re-entry revealed by simultaneous sub-epicardial and sub-endocardial optical mapping in explanted human hearts. *Eur. Heart J.* 36, 2390-2401 (2015).
7. Gomes,J.A., Hariman,R.I., & Chowdry,I.A. New application of direct sinus node recordings in man: assessment of sinus node recovery time. *Circulation* 70, 663-671 (1984).
8. Lou,Q. *et al.* Upregulation of adenosine A1 receptors facilitates sinoatrial node dysfunction in chronic canine heart failure by exacerbating nodal conduction abnormalities revealed by novel dual-sided intramural optical mapping. *Circulation* 130, 315-324 (2014).
9. Li,N. *et al.* Molecular Mapping of Sinoatrial Node HCN Channel Expression in the Human Heart. *Circ. Arrhythm. Electrophysiol.* 8, 1219-1227 (2015).

10. Csepe,T.A. *et al.* Human sinoatrial node structure: 3D microanatomy of sinoatrial conduction pathways. *Prog. Biophys. Mol. Biol.* 120, 164-178 (2016).

11. Fedorov,V.V., Glukhov,A.V., & Chang,R. Conduction barriers and pathways of the sinoatrial pacemaker complex: their role in normal rhythm and atrial arrhythmias. *Am. J. Physiol Heart Circ. Physiol* 302, H1773-H1783 (2012).

12. Chandler,N.J. *et al.* Molecular architecture of the human sinus node: insights into the function of the cardiac pacemaker. *Circulation* 119, 1562-1575 (2009).

13. El,R.M. *et al.* Protein Phosphatase 2A Regulates Cardiac Na(+) Channels. *Circ. Res.* 124, 737-746 (2019).

14. Radwanski,P.B. *et al.* Neuronal Na+ channel blockade suppresses arrhythmogenic diastolic Ca2+ release. *Cardiovasc. Res.* 106, 143-152 (2015).

15. Fedorov,V.V., Hucker,W.J., Dobrzynski,H., Rosenshtraukh,L.V., & Efimov,I.R. Postganglionic Nerve Stimulation Induces Temporal Inhibition of Excitability in the Rabbit Sinoatrial Node. *Am J Physiol* 291, H612-H623 (2006).

16. Fabbri,A., Fantini,M., Wilders,R., & Severi,S. Computational analysis of the human sinus node action potential: model development and effects of mutations. *J. Physiol* 595, 2365-2396 (2017).

17. Courtemanche,M., Ramirez,R.J., & Nattel,S. Ionic mechanisms underlying human atrial action potential properties: insights from a mathematical model. *Am. J. Physiol* 275, H301-H321 (1998).

18. Shaw,R.M. & Rudy,Y. Ionic mechanisms of propagation in cardiac tissue. Roles of the sodium and L-type calcium currents during reduced excitability and decreased gap junction coupling. *Circ. Res* 81, 727-741 (1997).
