# Supplementary material for: Impaired neuronal sodium channels cause intranodal conduction failure and reentrant arrhythmias in human sinoatrial node
Source: Nat Commun. 2020 Jan 24;11:512. doi: 10.1038/s41467-019-14039-8 (PMC6981137; doi:10.1038/s41467-019-14039-8)
Supplement: Supplementary file 3 — Description of Additional Supplementary Files [file 41467_2019_14039_MOESM3_ESM.docx]

**Description of Additional Supplementary Files**

**File Name: Supplementary Movie 1**

**Description:** SAN micro-reentry for Fig. 4b beat 1. Top, Optical field of view showing relative fluorescence with blue representing polarized myocardium and red representing depolarized myocardium. Center oval represents border of the sinoatrial node (SAN). Micro-reentry seen by following slow conduction from the center SAN moving towards the superior SAN, then returning back to the inferior SAN due to longitudinal conduction dissociation within the SAN. SAN conduction does not exit a sinoatrial conduction pathway to activate the surrounding right atrium (RA) leading to exit block conditions. Bottom, Optical action potentials from the SAN and RA at locations marked by asterisks of corresponding color in the above optical field of view. IVC indicates inferior vena cava region; RAA, right atrial appendage.

**File Name: Supplementary Movie 2**

**Description:** SAN macro-reentry for Fig. 4b beats 7 to 9. Top, Optical field of view showing relative fluorescence with blue representing polarized myocardium and red representing depolarized myocardium. Center oval represents border of the sinoatrial node (SAN). Macro-reentry seen by following slow conduction from the center SAN moving out the inferior lateral sinoatrial conduction pathway (SACP, right arrow) to activate the surrounding right atrium (RA). Conduction then moves from the right atrium to the inferior medial SACP (left arrow) to re-excite the SAN. Two macro-reentrant beats are shown. Bottom, Optical action potentials from the SAN and RA at locations marked by asterisks of corresponding color in the above optical field of view. IVC indicates inferior vena cava region; RAA, right atrial appendage.
