# Supplementary material for: Impaired neuronal sodium channels cause intranodal conduction failure and reentrant arrhythmias in human sinoatrial node
Source: Nat Commun. 2020 Jan 24;11:512. doi: 10.1038/s41467-019-14039-8 (PMC6981137; doi:10.1038/s41467-019-14039-8)
Supplement: Supplementary file 6 — Reporting Summary [file 41467_2019_14039_MOESM6_ESM.pdf]

nature research

Corresponding author(s): Dr. Vadim V Fedorov

Last updated by author(s): Nov 22, 2019

Reporting Summary

Nature Research wishes to improve the reproducibility of the work that we publish. This form provides structure for consistency and transparency in reporting. For further information on Nature Research policies, see Authors & Referees and the Editorial Policy Checklist .

Statistics

For all statistical analyses, confirm that the following items are present in the figure legend, table legend, main text, or Methods section.

n/a	Confirmed	
☐	☒	The exact sample size (*n*) for each experimental group/condition, given as a discrete number and unit of measurement
☐	☒	A statement on whether measurements were taken from distinct samples or whether the same sample was measured repeatedly
☐	☒	The statistical test(s) used AND whether they are one- or two-sided<br>*Only common tests should be described solely by name; describe more complex techniques in the Methods section.*
☐	☒	A description of all covariates tested
☐	☒	A description of any assumptions or corrections, such as tests of normality and adjustment for multiple comparisons
☐	☒	A full description of the statistical parameters including central tendency (e.g. means) or other basic estimates (e.g. regression coefficient) AND variation (e.g. standard deviation) or associated estimates of uncertainty (e.g. confidence intervals)
☐	☒	For null hypothesis testing, the test statistic (e.g. *F*, *t*, *r*) with confidence intervals, effect sizes, degrees of freedom and *P* value noted<br>*Give P values as exact values whenever suitable.*
☒	☐	For Bayesian analysis, information on the choice of priors and Markov chain Monte Carlo settings
☒	☐	For hierarchical and complex designs, identification of the appropriate level for tests and full reporting of outcomes
☒	☐	Estimates of effect sizes (e.g. Cohen's *d*, Pearson's *r*), indicating how they were calculated

Our web collection on statistics for biologists contains articles on many of the points above.

Software and code

Policy information about availability of computer code

Data collection	Optical mapping signals were collected using commercially available BV_MC05Exp_2IF Version 1.2 from BrainVision. Electrograms were recorded on LabChart version v8.1.11. A custom built C software was used to generated all computer simulations and has been described previously by our group (Zhao et al. Circulation: Arrhythmia and Electrophysiology 2012, 5 (2), 361-370) and others (Seemann and Zhang et al. 2006 https://doi.org/10.1098/rsta.2006.1781). A free version of this software (BeatBox) can be downloaded for academic use at http://empslocal.ex.ac.uk/people/staff/vnb262/projects/BeatBox/; Our utilized ionic model (Fabbri et al J. Physiol 2017, 595, 2365-2396) is freely available from the repository CellML (https://www.cellml.org/). Custom code and related files used for computer simulations in this study have been deposited at GitHub :https://github.com/Charcol97/Fabbri_HumanSAN_OSU
Data analysis	Optical mapping data were analyzed on a custom MATLAB program detailed in Fedorov et al. Heart Rhythm 2008;5:593– 604.<br>The open source program R 3.4.4 with packages emmeans and lme4 was used for all statistical analyses. Computational results were post processed with MATLAB (See Zhao et al. Journal Am Heart Assoc. 2017:6 (8), e005922).

For manuscripts utilizing custom algorithms or software that are central to the research but not yet described in published literature, software must be made available to editors/reviewers. We strongly encourage code deposition in a community repository (e.g. GitHub). See the Nature Research guidelines for submitting code & software for further information.

Data

Policy information about availability of data

All manuscripts must include a data availability statement. This statement should provide the following information, where applicable:

- Accession codes, unique identifiers, or web links for publicly available datasets
- A list of figures that have associated raw data
- A description of any restrictions on data availability

Raw source data underlying all reported averages in graphs and charts, and uncropped versions of blots presented in the figures are available as a Source Data file. All data are presented within the manuscript, the online data supplemental file, or are available upon reasonable request to the corresponding author.

Field-specific reporting

Please select the one below that is the best fit for your research. If you are not sure, read the appropriate sections before making your selection.

- [x] Life sciences
- [ ] Behavioural & social sciences
- [ ] Ecological, evolutionary & environmental sciences

For a reference copy of the document with all sections, see nature.com/documents/nr-reporting-summary-flat.pdf

Life sciences study design

All studies must disclose on these points even when the disclosure is negative.

Sample size	Sample sizes were determined by previous experience with human heart data.
Data exclusions	Some hearts were excluded from optical mapping analysis due to unstable SAN rhythm at control conditions.
Replication	Molecular samples, including qPCR data, was run in duplicate or triplicate to test consistency. During functional experiments, pacing and autonomic challenges were repeated in the same heart to test consistency.
Randomization	Hearts were randomized into either functional optical mapping group or molecular mapping group.
Blinding	Investigators were blinded to heart etiology, including detailed histories of co-morbidities and risk factors, until data collection was complete. Due to the human tissue collection process, failing vs non-failing status of the heart could not be blinded.

Reporting for specific materials, systems and methods

We require information from authors about some types of materials, experimental systems and methods used in many studies. Here, indicate whether each material, system or method listed is relevant to your study. If you are not sure if a list item applies to your research, read the appropriate section before selecting a response.

Materials & experimental systems

n/a	Involved in the study	
[ ]	[x]	Antibodies
[x]	[ ]	Eukaryotic cell lines
[x]	[ ]	Palaeontology
[x]	[ ]	Animals and other organisms
[x]	[ ]	Human research participants
[x]	[ ]	Clinical data

Methods

n/a	Involved in the study	
[x]	[ ]	ChIP-seq
[x]	[ ]	Flow cytometry
[x]	[ ]	MRI-based neuroimaging

Antibodies

Antibodies used	Anti-Nav1.5(custom-made in Dr. Peter Mohler's lab); anti-Nav1.6 (Alomone ASC-009); anti-connexin 43(Sigma c-6219);anti-Glyceraldehyde 3-phosphate dehydrogenase (Sigma G-8795);anti-α-actinin (Sigma A-7811); anti-vimentin (Sigma v-6389); anti-tyrosine hydroxylase (Millipore AB-152).
Validation	Antibodies including GAPDH, alpha actinin, Connexin 43 and Vimentin have been validated in our and other previous studies; Nav 1.5 and Nav 1.6 antibodies were first tested on positive and negative control tissues including mouse brain and skeletal muscle tissues.
